# Physical Activity and Psychological Well-Being During the COVID-19 Lockdown: Relationships With Motivational Quality and Nature Contexts

**DOI:** 10.3389/fspor.2021.637576

**Published:** 2021-02-25

**Authors:** Matthew Jenkins, Susan Houge Mackenzie, Ken Hodge, Elaine Anne Hargreaves, Jessica R. Calverley, Craig Lee

**Affiliations:** ^1^Department of Psychological Medicine, University of Otago, Wellington, New Zealand; ^2^Department of Tourism, University of Otago, Dunedin, New Zealand; ^3^School of Physical Education, Sport and Exercise Sciences, University of Otago, Dunedin, New Zealand

**Keywords:** physical activity, motivation, psychological well-being, nature, COVID-19

## Abstract

The COVID-19 pandemic is a global event that has already had substantive negative impacts on psychological well-being. This study investigated the relationship between physical activity (PA) and psychological well-being during a country-wide COVID-19 lockdown in New Zealand. Motivational quality and PA context (nature-based or non-nature-based) were included as potential mediating and moderating variables within this relationship, respectively. Participants completed an online survey assessing psychological well-being, weekly PA levels, and PA during the second and third weeks of the 7 week COVID-19 lockdown period in New Zealand. Data were analysed using Partial Least Squares Structural Equation Modelling. Results showed that PA significantly predicted psychological well-being, with no significant difference evident in psychological well-being dependent on whether PA was nature or non-nature-based. Nature-based PA was a stronger predictor of intrinsic motivation compared to non-nature-based PA, and intrinsic motivation was positively associated with psychological well-being. In contrast, non-nature-based PA was a stronger predictor of introjected regulation compared to nature-based PA, which was negatively associated with psychological well-being. Overall, these findings suggest that (1) weekly PA was associated with increased psychological well-being during the lockdown, and (2) nature-based PA may foster psychological well-being via effects on motivation. The implications for continued participation in PA will be discussed.

## Introduction

On January 30, 2020, the World Health Organisation (WHO) designated the COVID-19 outbreak a pandemic (World Health Organisation, 2020a). In New Zealand (NZ), the government declared a mandatory “Level 4 Lockdown” to control the virus spread, which lasted from March 25 to April 27, 2020. During this time period, residents were urged to stay at home unless undertaking a limited range of “essential” activities, including for certain types of employment (e.g., healthcare), shopping for essential groceries, medical reasons, and outdoor exercise or recreation. Further, people were required to maintain a physical distance of at least 2 metres from others outside their households. The restrictions imposed in the NZ lockdown are similar to those employed internationally.

The inclusion of exercise and recreation as a permissible outdoor activity (albeit in the vicinity of people's homes) was based on the New Zealand government's assertion that “exercise and recreation is an important part of maintaining our health and well-being,” with a particular emphasis on exercise being “good for mental health” (New Zealand Government, 2020, para 1). Thus, encouraging outdoor physical activity (PA) formed a part of the government's attempt to mitigate the potential for psychological distress related to COVID-19 events. Indeed, it was predicted that the mental health fallout from COVID-19 would include high levels of psychological distress arising from health concerns, social isolation or relationship friction, and long-term financial insecurity (Anderson, 2020). Research undertaken during the lockdown period in New Zealand (e.g., Every-Palmer et al., 2020) and globally (e.g., Roma et al., 2020; Vindegaard and Benros, 2020) supported these predictions providing evidence of increased depression, anxiety, and psychological distress.

Emphasising PA as a buffer against psychological distress and preserving psychological well-being stems from a robust evidence base, and has been advocated internationally as a way to reduce the negative impacts of the pandemic (World Health Organisation, 2020b). This raised the question of whether being physically active during lockdown influenced psychological well-being.

Psychological well-being is defined as the “personal perception and experience of positive and negative emotional responses and global and domain-specific cognitive evaluations of satisfaction with life” (Proctor, 2014, p. 275). Regular PA has been consistently associated with psychological well-being (Biddle et al., 2003), particularly leisure-time PA (White et al., 2017), as well as reduced psychological distress across the lifespan (e.g., Allison et al., 2005; George et al., 2012). Thus, the extant literature suggests that PA may have been a significant means of maintaining psychological well-being in the disruptive and potentially distressing context of a COVID-19 lockdown (Every-Palmer et al., 2020).

An important factor in whether an individual undertakes (and maintains) PA is the type of motivation that individual has for PA. Within the framework of self-determination theory (SDT; Ryan and Deci, 2017), motivation is described in terms of its quality, ranging from amotivation (a lack of motivation) to controlled and autonomous forms of motivation. In relation to PA, amotivation represents a motivational state wherein an individual sees no value in PA. Thus, they are likely to be either inactive or on the verge of discontinuing PA. Controlled motivation describes undertaking an activity out of obligation, either due to external rewards or punishments (known as *external regulation*) or internalised, self-imposed pressure (known as *introjected regulation*). In relation to PA, controlled motivation manifests when an individual is exercising because they feel they *have to* (e.g., to obtain a certain body image, to please significant others, or for other ego-based reasons; Ryan and Deci, 2017).

In contrast, autonomous motivation refers to an individual experiencing a sense of volition, choice, and self-endorsement when engaging in an activity or behaviour (Deci and Ryan, 2002). This could mean undertaking an activity for its own sake out of enjoyment or love (*intrinsic motivation*), or because the activity aligns with an individual's sense of self (*integrated regulation*) or personally-held values (*identified regulation*). Thus, autonomous motivation for a given behaviour is associated with more “authentic” living in the sense of behaving in line with one's personally chosen values (Deci and Ryan, 2008; Milyavskaya and Koestner, 2011; Hortop et al., 2013). Within the context of PA, autonomous motivation is consistently associated with long-term adherence to PA behaviour, whereas controlled motivation has been associated with higher levels of attrition (Teixeira et al., 2012).

Autonomous motivation is positively associated with psychological well-being while controlled motivation is negatively associated with psychological well-being (Briki, 2016), due to a perceived need to comply with external demands and subsequent feelings of guilt or shame if the behaviour in question is not undertaken (Ryan and Deci, 2017). To the authors' knowledge, the relationship between amotivation and psychological well-being has not been researched. However, because amotivation is characterised by a lack of control over one's own behaviour (Stavrou, 2008), it has been suggested to be associated with poor functioning and depressed symptoms (Briki, 2016) and thus is likely to have a negative relationship with psychological well-being.

During New Zealand's lockdown period, the government encouraged low-risk outdoor PA (New Zealand Government, 2020). This allowed for nature-based PA, defined as “any outdoor area with greenery or other natural features that contrast with the built environment” (Lackey et al., 2019, p. 2), encompassing a wide range of environments including forests, parklands, lakes, wetlands, and beaches. There is growing evidence that nature-based PA can confer additional benefits for psychological well-being beyond those conferred by PA alone. For example, one systematic review identified that nature-based PA provides synergistic psychological benefits beyond those attained from PA in non-natural environments (Thompson Coon et al., 2011). More recently, Eigenschenk et al.'s (2019) review identified a wide range of benefits specifically associated with nature-based PA, including: enhanced general well-being, psychological stability, life satisfaction, emotional intelligence, intellectual flexibility, mindfulness, empathy, self-esteem, self-actualisation, social capital, educational performance, and intrinsic motivation.

The potential influence of nature on motivational quality has also been recognised. Wolsko and Lindberg (2013) posited that spending time in nature ultimately reduces the influence of others on one's self-worth (controlled motivation) and instead cultivates internally-driven motives (autonomous motivation). Indeed, there is growing evidence that nature-based PA is associated with autonomous motivation, most notably in the form of intrinsic motivation (e.g., Gladwell et al., 2013; Eigenschenk et al., 2019) and that nature-based PA is more likely to be associated with autonomous motivation and adherence to PA compared to non-nature-based PA (Fraser et al., 2019).

Thus, there are both suggested and established associations amongst motivational quality, psychological well-being, and PA (in and away from nature). There is scope to explore these relationships during the events of COVID-19 in order to better understand how PA within different contexts may influence psychological well-being under times of psychological distress. During the COVID-19 lockdown, we might expect PA to have at least mitigated the psychological distress that we know was present during this period. In addition, nature-based PA may have conferred psychological benefits above and beyond non-nature-based PA via mechanisms related to motivational quality. We set out to examine the relationships amongst these variables.

Based on the literature reviewed, the aims of the present study were 2-fold. First, to investigate the relationship between PA, motivational quality and psychological well-being during the COVID-19 lockdown. Second, to investigate the potential influence of PA context (nature vs. non-nature based) on motivational quality and psychological well-being.

We had two research questions. During the NZ lockdown period: (1) Was there a relationship between weekly PA and psychological well-being? If so, was this relationship moderated by PA context (nature vs. non-nature)?; (2) Did motivational quality mediate the relationship between weekly PA and psychological well-being? If so, did this mediating role vary by PA context (nature vs. non-nature)? Hypothesised relationships amongst variables can be seen in Table 1.

**Table 1:** Hypothesised relationships amongst variables.

**Research question 1**	
Hypothesis (H) 1	Weekly PA will positively predict psychological well-being, and this relationship would be significantly stronger in a nature-based PA context compared to a non-nature-based PA context.
**Research question 2**	
H2	Amotivation will negatively mediate the relationship between weekly PA and psychological well-being.
H3	External regulation will negatively mediate the relationship between weekly PA and psychological well-being.
H4	Introjected regulation will negatively mediate the relationship between weekly PA and psychological well-being.
H5	Identified regulation will positively mediate the relationship between weekly PA and psychological well-being.
H6	Integrated regulation will positively mediate the relationship between weekly PA and psychological well-being.
H7	Intrinsic motivation will positively mediate the relationship between weekly PA and psychological well-being.
H8	The structural relationships among the constructs in the model will be stronger in a nature-based PA context, compared to the non-nature-based PA context.

## Materials and Methods

### Participants and Sampling

Participants were sought across the general NZ population. The inclusion criteria stipulated that participants must be aged 18 years or older and living in NZ for the duration of the lockdown period. Participants were excluded if they had any contraindications (e.g., illness, injury) that prevented PA during the lockdown period. These criteria were assessed via screening questions at the beginning of the survey. A convenience sample was recruited using social media recruitment methods (Akers and Gordon, 2018) and a virtual snowball recruitment technique (Baltar and Brunet, 2012). Study information was shared via Facebook and circulated via email to contacts at large universities and other NZ organisations (e.g., regional and national sports organisations, city councils) for dissemination through their networks. This study comprises part of a larger project investigating determinants and outcomes of PA as a result of the COVID pandemic.

A cross-sectional survey, hosted by the online survey platform Qualtrics, was conducted for 8 days between the second and third weeks (April 8 to April 15, 2020) of NZ's Level 4 lockdown period. The full project survey took an average of 12 min to complete. Ethical approval was obtained prior to data collection from a university ethics committee (redacted for peer review; reference: D20/214). All participants provided online informed consent before completing the survey. The survey elicited 858 responses. Following data cleaning, which omitted cases with too much missing data (<5% missing data for each variable; 99 cases), data from 759 participants were analysed.

### Survey Measures

The survey measured weekly PA, nature-based PA, psychological well-being, and motivational quality in relation to PA, using the instruments below.

#### Weekly PA Levels

The International Physical Activity Questionnaire-Short Form (IPAQ-SF; Craig et al., 2003) is a 7-item measure of self-reported PA, measuring the amount of moderate- and vigorous-intensity PA, walking, and sitting undertaken by participants over the previous 7 days. Example items include: “During the last 7 days, on how many days did you do vigorous activities like heavy lifting, exercise classes, or fast cycling for at least 10 min at a time?” followed by the question: “How much time did you usually spend doing vigorous physical activities on one of those days?” The IPAQ-SF has demonstrated good validity and consistency (e.g., Lee et al., 2011).

#### Nature-Based PA

Participants were asked if they had the opportunity to be physically active in nature (“Do you currently have the option to be active in or around natural environments?”). Participants responding with “yes” were then asked: “In the past 7 days, have you participated in any nature-based physical activity?”.

#### Psychological Well-Being

The World Health Organisation Well-Being Index (WHO-5; World Health Organisation, 1998) is a self-report measure of subjective psychological well-being, containing five items scored on a Likert-type scale ranging from 0 (at no time) to 5 (all of the time). The item “Thinking about yourself and how you have felt over the last 7 days, to what extent have you felt…” is followed by items that include: “I woke up feeling fresh and rested” and “I have felt calm and relaxed.” The WHO-5 has been shown to have high reliability and validity across many different samples in different countries (Topp et al., 2015).

#### Motivational Quality

The Behavioural Regulation in Exercise (BREQ-3 PA version; Markland and Tobin, 2004) was used to measure motivation for PA. The BREQ-3 contains 24 items, each scored on a 5-point Likert-type scale from 0 (not true for me) to 4 (very true for me). Example autonomous motivation items include: “I am physically active because I enjoy it” (intrinsic motivation), “It's important to me to be regularly physically active” (identified regulation) and “I consider physical activity as part of my identity” (integrated regulation). Controlled items include: “I feel ashamed when I miss a physical activity session” (introjected regulation) and “I take part in physical activity because my friends/family/partner say I should” (external regulation). The measure also includes a subscale pertaining to a lack of motivation for PA (“I do not see the point in being physically active”). Previous research has shown that the BREQ-3 has high reliability and validity (MacDonald's ω = 0.93) and its six-factor structure has been supported (e.g., Jenkins et al., 2020).

### Data Cleaning

For the IPAQ-SF, data screening, cleaning, and coding were undertaken according to Craig et al.'s (2003) detailed guidelines. This included the truncation of data points indicating more than 960 min of PA per week as these are suggested to be outliers (Craig et al., 2003). Very low missing data were present for the items used in the structural model (<5% missing data for each variable), and the Little's Missing Completely at Random test produced a non-significant result, meaning data was missing completely at random. Thus, any missing data was estimated using the Expectation-Maximisation algorithm (Peters and Enders, 2002).

### Data Analysis

Partial Least Squares Structural Equation Modelling (PLS-SEM) using SmartPLS (v. 3.2.7) (Ringle et al., 2018) was used for data analysis. PLS-SEM was used over covariance-based SEM (CB-SEM) due to the main objective of this study being the prediction of psychological well-being instead of theory testing and model validation (which is usually the focus of CB-SEM) (Hair et al., 2017). In addition, there was evidence of some non-normality in our data (see **Table 3**, skewness and kurtosis), and PLS-SEM is useful in such situations as it is a non-parametric method that can tolerate mild departures from normality within the data (Hair et al., 2017). Also, PLS-SEM allows issues with regard to sample and sub-sample size limitations to be overcome when dealing with complex models and moderation analysis. Thus, PLS-SEM was used as the model to be estimated was complex relative to sample size, comprising 11 latent constructs with multiple indicators (Haenlein and Kaplan, 2004). In addition, PLS-SEM is more sensitive than CB-SEM in detecting moderator effects given its effectiveness in dealing with measurement error, which also functions to decrease sample size requirements (Lowry and Gaskin, 2014).

### Sample Size Adequacy

A priori and *post-hoc* power analyses using G^*^Power was used to determine the adequacy of the sample size (Faul et al., 2009). Using a suggested minimum *R*^2^ value of 0.10, a statistical power of 95%, and nine predictors (the psychological well-being construct has the largest number of predictors) (Cohen, 1988), the a priori G^*^ Power calculation indicated that a minimum sample size of 211 would be required. In addition, the *post-hoc* G^*^ Power calculation for a minimum *R*^2^ of 0.10, a sample size of 759 (the number of usable responses obtained), and nine predictors indicated that the statistical power achieved using the study's sample size was 1.0, which is well above Cohen's (1988) recommendations, thus, justifying the adequacy of our sample size.

### Common Method Bias

Common method bias (CMB) was mitigated through *post-hoc* analysis following Podsakoff et al.'s (2003) recommendations. We applied the marker variable approach using the steps designed for PLS-SEM (Rönkkö and Ylitalo, 2011). Specifically, we estimated the structural model with and without a marker construct as an exogenous variable predicting each construct. Firstly, the correlations between the marker construct and all constructs in the model were very low (correlations ranging between −0.13 and 0.07), and its effect on all constructs were non-significant. Secondly, comparing of results between the structural models, with and without, the marker construct showed no notable differences, and all theorised paths maintained similar path estimates and levels of statistical significance. Thus, the *post-hoc* analysis does not suggest a threat of CMB for this study (Podsakoff et al., 2003; Rönkkö and Ylitalo, 2011).

## Results

We first present the descriptive statistics obtained from our sample, followed by the measurement models (the outer model analysis), including internal and indicator reliability, and convergent and discriminant validity of the constructs in our sample. We then describe the relationships amongst the variables in terms of mediation and moderation analysis (inner model analysis) in relation to our research questions and hypotheses.

### Descriptive Statistics of the Sample

Participants' mean, and median, age was 43 years (range = 18–81 years, sd = 13.71). They were predominantly employed (90.5%), female (73%), and NZ European (82.9%; 0.9% identified as Māori; 0.6% identified as Chinese; 13.2% identified as Other). Essential workers comprised 13.8% of respondents. Many participants had some form of postgraduate education (46.4%) and approximately half were married (50.2%). Descriptive statistics, including mean scale scores, can be seen in Table 2.

**Table 2:** Descriptive statistics of the sample.

**Variable**	**Category**	***N* (%)**
Gender	Male	195 (26.2)
	Female	544 (73.0)
	Gender diverse	3 (0.4)
	Prefer not to say	3 (0.4)
Education	No formal qualification	10 (1.3)
	Less than high school	7 (0.9)
	High school graduate	32 (4.3)
	Some university / tertiary	53 (7.1)
	Certificate or diploma	90 (12.1)
	University undergraduate degree	208 (27.9)
	Postgraduate and above	345 (46.3)
Ethnicity	New Zealand European	574 (82.9)
	Māori	6 (0.9)
	Samoan	1 (0.1)
	Cook Island Māori	2 (0.3)
	Chinese	4 (0.6)
	Indian	2 (0.3)
	Other	91 (13.2)
	Prefer not to say	12 (1.7)
Employment	Yes	674 (90.5)
	No	71 (9.5)
Essential worker	Yes	102 (13.8)
	No	638 (86.2)
Relationship status	Single	140 (21.8)
	Married	381 (50.2)
	Divorced	14 (2.2)
	De facto partnership	94 (14.7)
	Civil partnership	3 (0.5)
	Prefer not to say	9 (1.4)
**Age**		
Mean = 43.04; S.D. = 13.71, Median = 43, Mode = 55, Lowest age = 18, Highest age = 81		
**Amotivation**		
Mean = 0.16, S.D. = 0.507		
**External Regulation**		
Mean = 0.65, S.D. = 0.938		
**Introjected Regulation**		
Mean = 2.14, S.D. = 1.263		
**Identified Regulation**		
Mean = 3.35, S.D. = 0.853		
**Integrated Regulation**		
Mean = 2.76, S.D. = 1.252		
**Intrinsic Motivation**		
Mean = 3.09, S.D. = 0.969		
**Psychological Well-Being**		
Mean = 58.06, S.D. = 24.69		

### Outer Model Analysis: Assessing the Measurement Models

Internal reliability of all constructs was established with Cronbach's α and Composite Reliability values >0.7 (Hair et al., 2017) (see Table 3). Indicator reliability for all constructs was established as all indicators had outer loadings >0.6. Convergent validity of all constructs was established as all AVE values >0.5. Discriminant validity of all constructs was established as the confidence intervals for the Heterotrait-Monotrait ratio (HTMT) of the correlations among all latent constructs did not include 1 (Hair et al., 2017).

**Table 3:** The (outer) structural model.

**Latent construct**	**Indicators**	**Mean**	**S.D**.	**Skew**	**Kurtosis**	**Outer loadings**	**Cronbach's α**	**Composite reliability**	**Average**	**HTMT**	***R^**2**^***	***Q^**2**^***
Amotivation (AMOT)	AMOT1	0.19	0.545	3.560	14.444	0.823	0.847	0.897	0.685	Does not include 1	0.082	0.054
	AMOT2	0.18	0.533	3.432	12.753	0.852						
	AMOT3	0.15	0.493	4.288	22.236	0.816						
	AMOT4	0.12	0.455	4.604	24.788	0.821						
External regulation (EXT)	EXT1	0.95	1.053	0.821	−0.212	0.866	0.872	0.909	0.715	Does not include 1	0.013	0.007
	EXT2	0.63	0.941	1.447	1.464	0.873						
	EXT3	0.45	0.828	2.039	4.057	0.79						
	EXT4	0.58	0.928	1.639	2.164	0.852						
Introjected regulation (INTRO)	INTRO1	2.73	1.146	−0.593	−0.444	0.835	0.854	0.899	0.691	Does not include 1	0.031	0.018
	INTRO2	1.65	1.270	0.207	−1.011	0.807						
	INTRO3	1.88	1.354	0.088	−1.137	0.828						
	INTRO4	2.31	1.281	−0.262	−0.964	0.855						
Identified regulation(IDENT)	IDENT1	3.47	0.808	−1.509	1.918	0.88	0.848	0.898	0.687	Does not include 1	0.181	0.121
	IDENT2	3.60	0.716	−1.948	4.042	0.811						
	IDENT3	3.55	0.704	−1.569	2.503	0.847						
	IDENT4	2.78	1.184	−0.713	−0.368	0.774						
Integrated regulation (INTEG)	INTEG1	2.93	1.126	−0.859	−0.095	0.801	0.907	0.936	0.785	Does not include 1	0.194	0.151
	INTEG2	2.53	1.400	−0.542	−1.012	0.923						
	INTEG3	2.61	1.381	−0.616	−0.918	0.922						
	INTEG4	2.98	1.102	−0.975	0.245	0.892						
Intrinsic motivation (INTRIN)	INTRIN1	2.85	1.044	−0.720	0.115	0.891	0.938	0.955	0.842	Does not include 1	0.184	0.154
	INTRIN2	3.17	0.919	−1.003	0.659	0.933						
	INTRIN3	3.06	0.984	−0.961	0.616	0.934						
	INTRIN4	3.26	0.930	−1.274	1.342	0.913						
Psychological well–being (PWB)	PWB1	64.90	20.98	−0.832	0.167	0.83	0.859	0.896	0.632	Does not include 1	0.19	0.100
	PWB2	62.48	22.86	−0.642	−0.218	0.796						
	PWB3	52.96	25.51	−0.218	−0.864	0.808						
	PWB4	50.75	28.14	−0.151	−0.967	0.751						
	PWB5	59.21	25.98	−0.242	−0.829	0.787						

### Inner Model Analysis: Direct, Indirect, and Moderation Analysis

There were no collinearity issues among the predictor constructs with all VIF values <5 (Hair et al., 2017). The results of the direct and indirect effects of the exogenous variables on the endogenous variables are shown in Table 4. In terms of the model's predictive relevance, the Stone-Geisser *Q*^2^ values obtained through the blindfolding procedure (omission distance = 7) for the endogenous constructs of amotivation, external regulation, introjected regulation, identified regulation, integrated regulation, intrinsic motivation, nature relatedness, and psychological well-being were >0, supporting the predictive relevance of the model (Hair et al., 2017) (Table 4). Finally, due to evidence for differences in motivation across gender (e.g., Lauderdale et al., 2015) and age (e.g., Brunet and Sabiston, 2011), we controlled for these variables in the current analysis. Due to the fact that this was an exploratory study that was focused on isolating the effects of nature-based PA and motivation on the baseline model, other demographic variables (education, ethnicity, employment status, relationship status) were not controlled for within the analysis. Further, there was no theoretical grounding for differences amongst these demographic variables.

**Table 4:** Inner model analysis (baseline model).

**Path estimates of baseline model**	**Path coefficient**	**Effect size (*f*^**2**^)**	**95% CI**
**Weekly PA Total → Amotivation**	**−0.29**	**0.089**	**−0.34 to** **−0.24**
**Weekly PA Total → External Regulation**	**−0.12**	**0.014**	**−0.19 to** **−0.04**
**Weekly PA Total → Introjected Regulation**	**0.18**	**0.032**	**0.083–0.258**
**Weekly PA Total → Identified Regulation**	**0.43**	**0.221**	**0.37–0.48**
**Weekly PA Total → Integrated Regulation**	**0.44**	**0.241**	**0.38–0.50**
**Weekly PA Total → Intrinsic Motivation**	**0.43**	**0.225**	**0.38–0.483**
**Weekly PA Total → Psychological Well-Being**	**0.17**	**0.028**	**0.09–0.25**
Amotivation → Psychological Well-Being	0.06	0.003	−0.03–0.15
External Regulation → Psychological Well-Being	0.01	0	−0.07–0.08
**Introjected Regulation → Psychological Well-Being**	**−0.17**	**0.03**	**−0.25 to** **−0.09**
Identified Regulation → Psychological Well-Being	0.09	0.00	−0.061–0.23
Integrated Regulation → Psychological Well-Being	0.10	0.00	−0.04–0.24
**Intrinsic Motivation → Psychological Well-Being**	**0.14**	**0.01**	**0.01–0.26**
Weekly PA Total → Introjected Regulation → Psychological Well-Being^*^	**−0.03**	n/a	**−0.05 to** **−0.01**
Weekly PA Total → Intrinsic Motivation → Psychological Well-Being^*^	**0.06**	n/a	**0.01–0.12**

* *For brevity, only the indirect effects that were significant for the baseline model are presented in this table. Significant coefficients in **bold***.

**Figure 1:**
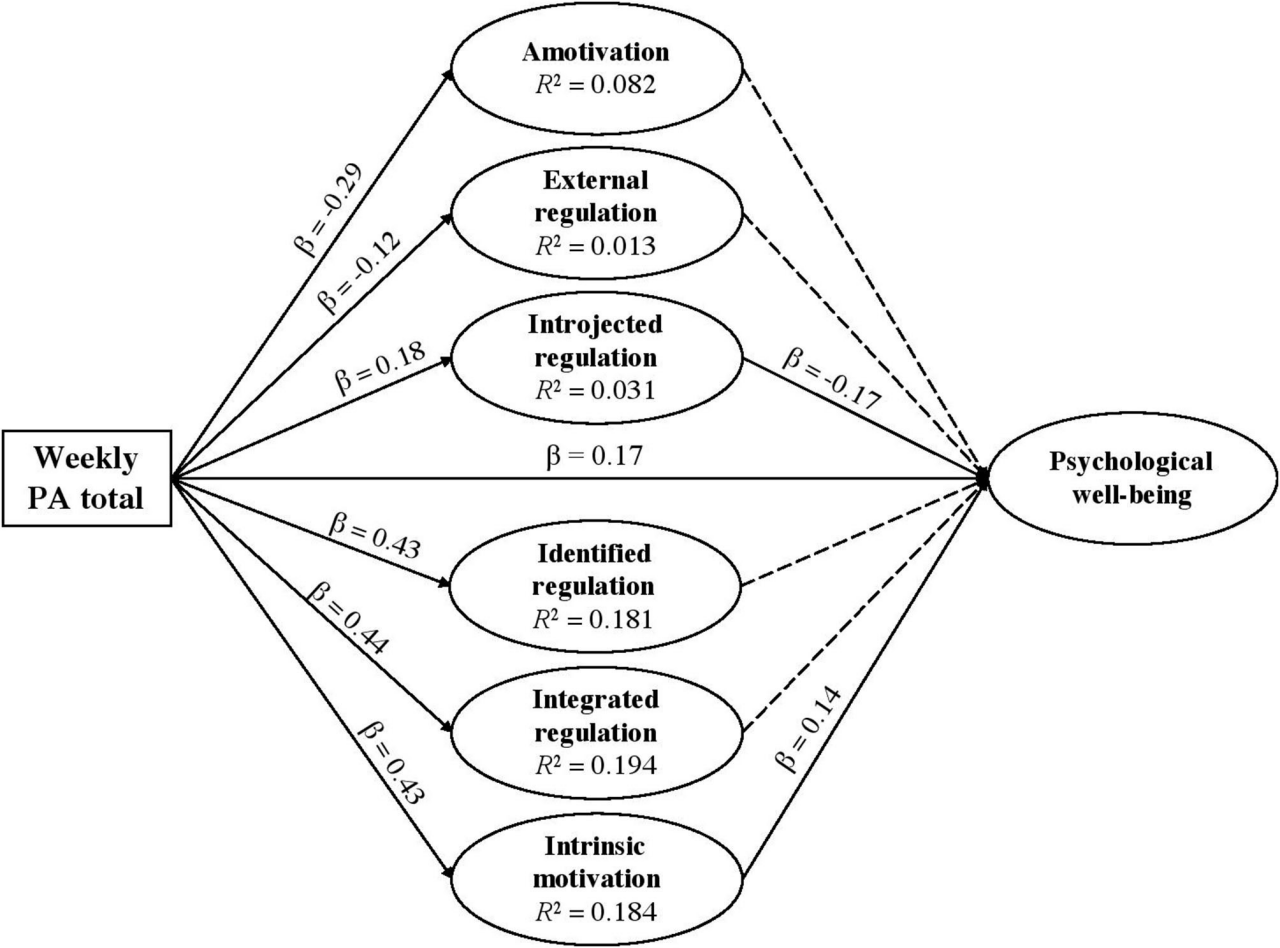
Path coefficients amongst variables. Solid lines represent significant paths. Dashed lines represent non-significant paths.

The structural model (see Figure 1) was cross-validated across the moderating variable of nature-based PA, meaning, whether the respondent had engaged in PA that was nature-based (*N* = 520) or not (*N* = 239), through Partial Least Squares Multi Group Analysis (PLS-MGA). Multi group permutation tests were used to assess whether there were significant differences in the path estimates between participants who undertook nature-based PA and those who undertook non-nature-based PA. These between-group differences are described in relation to their associated research questions (see Table 5).

**Table 5:** Multi group analysis results.

**Path estimate**	**Path coefficient for non-nature-based PA (*N* = 239)**	**Path coefficient for nature-based PA (*N* = 520)**
Weekly PA Total → Amotivation	−0.35^***^	−0.22^***^
Weekly PA Total → Identified Regulation	0.53^***^	0.34^***^
Weekly PA Total → Introjected Regulation	0.31^***^	0.10 ^n.s.^
Weekly PA Total → Introjected Regulation → Psychological Well-Being	−0.072^**^	−0.015 ^n.s.^

** Path estimate significant at p < 0.01;

*** *Path is significant at p < 0.001. Only path estimates that were significantly different between the two groups are presented in this table. n.s., non-significant*.

#### Research Question 1: Was There a Relationship Between Weekly PA and Psychological Well-Being? Was This Relationship Moderated by PA Context (Nature vs. Non-nature)?

Weekly PA significantly predicted psychological well-being (β = 0.17, effect size = 0.028, 95% confidence interval (CI) = 0.09–0.25). However, PA context did not moderate this relationship. Thus, hypothesis 1 was partially supported. Relevant results are shown in Table 4 and Figure 1.

#### Research Question 2: Did Motivational Quality Mediate the Relationship Between Weekly PA and Psychological Well-Being? If So, Did This Mediating Role Vary by PA Context (Nature vs. Non-nature)?

##### Controlled Forms of Motivation

Results demonstrate that amotivation and external regulation *did not* mediate the relationship between weekly PA and psychological well-being. Thus, hypotheses 2 and 3 were rejected. Results supported hypothesis 4 in showing that introjected regulation negatively mediated the relationship between weekly PA and psychological well-being (indirect path estimate = −0.03, 95% CI = −0.05 to −0.01). Relevant results are shown in Table 4 and Figure 1.

##### Autonomous Forms of Motivation

Results demonstrate that neither identified regulation nor integrated regulation mediated the relationship between weekly PA and psychological well-being. Thus, hypotheses 5 and 6 were rejected. Results supported hypothesis 7 in showing that intrinsic motivation positively mediated the relationship between weekly PA and psychological well-being (indirect path estimate = 0.06; 95% CI = 0.01–0.12). Relevant results are shown in Table 4 and Figure 1.

##### Moderation of Relationships by Context

Results demonstrate that only in a non-nature-based context did introjected regulation significantly mediate the relationship between weekly PA and psychological well-being. Further, the direct relationships between weekly PA and amotivation, identified regulation, and introjected regulation were significantly stronger within non-nature-based PA (see Table 5). There was no other moderating effect of PA context. Thus, hypothesis 8 was partially supported.

## Discussion

This study investigated the relationship between PA and psychological well-being during the NZ COVID-19 lockdown period, and whether motivational quality had a mediating role in this relationship. Further, we examined whether PA context (nature or non-nature based) moderated relationships amongst PA, motivational quality, and psychological well-being. As expected, the results indicated that PA was positively associated with psychological well-being. Contrary to predictions, the PA context did not directly influence the relationship between PA and psychological well-being in this sample.We also partially confirmed our hypothesis that motivational quality would mediate the relationship between PA and psychological well-being. Specifically, introjected regulation (a controlled form of motivation) negatively mediated the relationship between PA and psychological well-being, but only in non-nature-based contexts. Intrinsic motivation positively mediated the relationship between weekly PA and psychological well-being, but unexpectedly there was no significant difference between PA contexts.

### PA and Psychological Well-Being

These results support the volume of literature showing the important positive relationships between PA and psychological well-being (e.g., Biddle et al., 2003; Pretty et al., 2007; Stubbs et al., 2017). Our study extends this literature by showing that this relationship holds during periods of highly restricted mobility and associated psychological distress due to COVID-19 lockdowns.

### The Mediating Role of Motivational Quality

Hypotheses regarding relationships amongst weekly PA, motivational quality, and psychological well-being were partially supported. Contrary to predictions, neither amotivation, external, identified, nor integrated regulations significantly mediated the relationship between PA and psychological well-being. However, intrinsic motivation was a significant positive mediator and introjected a significant negative mediator of the relationship between PA and psychological well-being, reflecting previous research (Briki, 2016). Although we cannot infer causation from these data, this supports the notion that PA may affect psychological well-being via these types of motivation. According to these findings, if an individual was physically active on the basis of ego or guilt, then they were less likely to experience positive psychological well-being. Conversely, if an individual was physically active on the basis of enjoyment or love of the PA activity itself, they were more likely to experience psychological well-being. It should be noted, however, that effect sizes were significant-yet-small, and so we should be cautious when interpreting these results.

### The Role of PA Context

The relationships between PA and both amotivation and identified regulation were significantly stronger within non-nature-based contexts, as compared to nature-based contexts. However, again, these significant effect sizes remained small, so we should be circumspect in our interpretations. The finding regarding identified regulation was unexpected, as we predicted that all forms of autonomous motivation would be more likely within nature-based contexts. One potential explanation is that because identified regulation is based on motivation for relatively tangible and valued outcomes (e.g., improved health), individuals may feel those outcomes are more readily achieved, or are more obvious through non-nature-based PA.

As hypothesised, we found that non-nature-based PA predicted introjected regulation to a significantly greater degree than nature-based PA. This is noteworthy as introjected regulation (which is characterised by ego-based motives) negatively predicted psychological well-being. While causality cannot be inferred from our data, these findings align with Wolsko and Lindberg's (2013) hypothesis that ego-based motivations may decrease as a result of being in nature. Thus, future research should directly examine whether natural environments may reduce ego-oriented motivations for PA.

Many of the study findings regarding the role of nature-based contexts were unexpected considering the growing body of literature indicating that nature-based PA benefits psychological well-being in unique and synergistic ways beyond the benefits accrued by non-nature-based PA (Gladwell et al., 2013). Assessing how *often* people engaged in nature-based PA and whether they were active in *both* nature and non-nature-based contexts may have provided important additional data to better understand these relationships. It is possible that either a) people were not accessing nature often enough to generate additional psychological well-being benefits, or b) people sought alternative PA methods that provided comparable psychological well-being benefits. For example, reports indicated a global rise in the popularity of yoga as a form of PA during COVID-19 lockdowns (Merchlinksy, 2020), which is a predominantly indoor practice that is consistently associated with higher levels of psychological well-being (Brinsley et al., 2020). Past studies comparing nature-based and non-nature-based PA have overlooked practices such as yoga, in favour of comparisons between activities such as running and walking. Therefore, future research comparing nature and non-nature-based PA should expand the breadth of activities examined to provide more nuanced information regarding the importance of PA context for psychological well-being.

The finding that PA context did not significantly moderate the relationships amongst PA context, intrinsic motivation and psychological well-being was unexpected, because enjoyment of nature-based PA was one of the more robust findings in the extant literature (e.g., Eigenschenk et al., 2019). The effect of physical distancing requirements during the NZ lockdown may have influenced these results. In NZ, people were asked to maintain a two-metre distance from others at all times. This constant need to ensure physical distance from others may have introduced a novel source of anxiety that is not usually present in nature-based PA, leading to decreased enjoyment in such natural settings. For example, many nature-based walking tracks in NZ are too narrow to maintain a two-metre distance when passing others. In addition, the phenomenon of “exercise shaming” people who were perceived as being too physically active outdoors during lockdown periods may have influenced these findings (e.g., Graham-McLay, 2020). These unique factors may have offset any additional potential positive effects of nature-based PA by shifting people's focus from the natural environment to potential anxiety regarding violations of lockdown restrictions and/or the need to avoid other people, respectively. More research is needed to explore these conjectures as these potential explanations are speculative.

### Implications

Considering the main finding that PA was significantly associated with psychological well-being, a primary implication of this research is that PA should be promoted and supported during potentially stressful events, such as lockdowns. From a public health perspective, government policies should encourage and enable a wide range of PA during lockdowns and, where possible, accommodate opportunities for people to engage in nature-based PA. It is also clear that people should not be enticed to engage in PA on the basis of ego- or guilt-based forms of motivation, but rather encouraged to engage in forms of PA that create intrinsic motivation.

The findings also have theoretical implications for SDT in terms of demonstrating significantly different relationships amongst PA and specific types of motivation. In recent years, there has been a trend towards dichotomously grouping motivation into either *autonomous* or *controlled* categories. However, SDT scholars continue to debate whether this is the most appropriate approach to investigating or understanding motivations (e.g., Howard et al., 2020). The current results support a more fine-grained approach to examining motivation, rather than using an autonomous vs. controlled dichotomy. Although, one aspect that we did not consider was the *interaction* between different types of motivation. Individuals rarely have just one motive for undertaking PA and likely experience a mix of reasons. Approaches such as latent profile analyses (e.g., Lindwall et al., 2017) have the potential for greater insights into the relationships amongst motivation, psychological well-being, and PA context.

### Limitations and Strengths

The study provided cross-sectional insights into relationships between PA, nature, motivation, and psychological well-being in a unique life situation. However, this uniqueness also represents a limitation, in that the results may not generalise to life situations outside of a pandemic-induced lockdown. We also could not compare our results with pre-pandemic data, as these variables are not routinely collected. In addition, given the cross-sectional design, causal relationships cannot be inferred. Further, as previously mentioned, participants did not report the *amount* of nature-based PA undertaken. Thus, we cannot speculate on the relationships between dose of nature-based PA and motivation or psychological well-being. For instance, participants may have engaged in a single, 20-min bout of nature-based PA during the week, while the rest of their PA may have been non-nature-based. Understanding the extent to which participants engaged in nature-based PA would facilitate more nuanced data analysis and an evaluation of a “dose-response” effect for nature-based PA.

Finally, the obtained sample was not representative of the general NZ population. For example, a high proportion of participants were physically active (81.2% met weekly PA guidelines, compared to 50.8% in the general population; New Zealand Ministry of Health, 2019), and were predominantly highly-educated females. This limits the generalisability of the findings as we cannot assume that they would apply to other populations. Indeed, there may have been a ceiling effect in relation to PA. High PA levels may have reduced variation in the sample, thereby obscuring some of the relationships amongst PA, motivation and psychological well-being.

### Future Research

We suggest a number of future research avenues. First, to compare the psychological well-being outcomes of lockdowns that allow outdoor or nature-based PA (e.g., NZ) vs. those that did not (e.g., Spain). It will also be important to examine whether the relationships reported here remain in periods of increased mobility (e.g., under decreased, post-COVID-19 restrictions). For example, we speculated that the absence of a direct positive relationship between nature-based PA and psychological well-being may have been due to potential anxiety experienced when encountering others while engaging in nature-based PA. Future research could examine how these relationships alter once COVID-19 is no longer considered a widespread threat. In addition, longitudinal designs and time-lagged analysis are needed to directly examine potential causal relationships amongst the key variables. There is also a need to further examine the distinct roles of intrinsic motivation and introjected regulation, respectively, in relation to PA in “non-pandemic” circumstances, either cross-sectionally or via intervention designs.

## Conclusion

During a period of COVID 19 related lockdown restrictions, being physically active benefitted participants' psychological well-being. Ensuring individuals are intrinsically motivated for PA may provide additional benefit to psychological well-being, while individuals who have introjected regulation may experience lower psychological well-being when they exercise in non-nature-based PA contexts. These motivational associations between PA and psychological well-being suggested people may benefit from engaging in nature-based PA during lockdown periods, although more research is required to confirm this conclusion. Given the increasing frequency of pandemics in recent decades and expectations that this trend will continue (Ross et al., 2015), this research provides valuable insights regarding the relationships amongst PA, the context under which it is undertaken, motivational quality and psychological well-being during these periods.

## Data Availability Statement

The datasets presented in this study can be found in online repositories. The names of the repository/repositories and accession number(s) can be found in the article/supplementary material.

## Ethics Statement

The studies involving human participants were reviewed and approved by University of Otago Ethics Committee (ref. D20/214). The patients/participants provided their written informed consent to participate in this study.

## Author Contributions

MJ, EH, SH, KH, and JC conceived the study. MJ, JC, and EH coordinated data collection. MJ, JC, and CL cleaned and analysed the data. MJ led the manuscript writing and all authors contributed to the final submission.

## Conflict of Interest

The authors declare that the research was conducted in the absence of any commercial or financial relationships that could be construed as a potential conflict of interest.
